# Case Report: Peripheral Ulcerative Keratitis in Systemic Solid Tumour Malignancies

**DOI:** 10.3389/fmed.2022.907285

**Published:** 2022-05-31

**Authors:** Valencia Hui Xian Foo, Jodhbir Mehta, Anita Sook Yee Chan, Hon Shing Ong

**Affiliations:** ^1^Singapore National Eye Centre, Singapore, Singapore; ^2^Singapore Eye Research Institute, Singapore, Singapore; ^3^Ophthalmology and Visual Science Academic Clinical Research Program, Duke-National University of Singapore (NUS) Medical School, Singapore, Singapore

**Keywords:** malignancy, paraneoplastic, corneal, inflammation, peripheral ulcerative keratitis

## Abstract

**Purpose:**

To describe a case series of peripheral ulcerative keratitis (PUK) as a paraneoplastic condition in three patients with known underlying systemic solid tumor malignancies.

**Observations:**

Three patients with different systemic malignancies (1 recurrent breast cancer, 1 metastatic thyroid cancer, and 1 metastatic gastric adenocarcinoma) were identified to have PUK with significant corneal stromal melt. Autoimmune and infective work up for other etiologies were all negative. They all responded well to topical steroids and intravenous methylprednisolone. One patient had recurrences of her PUK and required repeated amniotic grafts and tectonic keratoplasties before her corneal condition stabilized.

**Conclusions and Importance:**

PUK can be a rare manifestation of systemic solid tumor malignancies. Although PUK may not be an indicator of progression of the underlying malignancy, it can be sight-threatening. This case series highlights the necessity for clinicians to refer patients with systemic malignancies presenting with inflamed eyes for an early ophthalmological review. This facilitates the detection of this blinding disease, allowing for early therapeutic interventions and potentially better visual outcomes for these patients.

## Introduction

Peripheral ulcerative keratitis (PUK) is a form of corneal stromal inflammation which involves crescentic sectoral thinning of the juxta-limbal cornea. In PUK, a local destructive process of the cornea is triggered by autoantibodies against a corneal-specific antigen that mediates the release of collagenolytic and proteolytic enzymes. This results in corneal stromal melting (1–3). Additionally, the release of proinflammatory cytokines further accelerates the collagen matrix destruction process (1). The incidence of PUK is estimated to be ~3 per million per year (4). Although rare, delays in its management may lead to progressive corneal stromal melt. Visual loss occurs from corneal thinning and scarring, and in severe cases, corneal perforation. Rates of corneal perforations in PUK have been reported to be between 15 and 35% (5, 6), with up to 10% of eyes requiring enucleation (6). More importantly, as PUK can be associated mainly with several potentially life-threatening autoimmune such as Rheumatoid Arthritis or Polyarthritis Nodosa or systemic infectious diseases (7), a timely and meticulous clinical work-up is of utmost importance.

Systemic malignancies have been reported as rare associations of PUK (8–10). Such reported associations are mostly hematological malignancies and include acute myeloid leukemia (11), chronic myeloid leukemia (CML) (8, 9), and acute lymphocytic leukemia (10). In these reports, most of the patients presented with PUK which subsequently led to the diagnosis of a hematological malignancy (9–11). Only one case report described a patient with PUK and a known diagnosis of CML (8). In all cases of hematological malignancies, the PUK resolved with topical immunosuppression and concurrent treatment of their underlying malignancies. A case of sebaceous gland carcinoma of the orbit has also been reported as the underlying etiology of PUK (9). In this case report, the eye required orbital exenteration. However, there are no reports of systemic solid organ malignancies in association with PUK in current literature.

We describe a case series of three patients with underlying systemic solid tumor malignancies who presented with PUK a few months after their initial diagnoses of malignancies were made. The pathophysiology of PUK in solid tumor malignancies is not well-understood; it could be postulated to be due to deposited immunocomplexes in the cornea limbus as a result of autoantibodies secretion from a solid tumor malignancy. Interestingly in our series, the original sites of patients' underlying primary solid organ malignancies were all distant from the eye. This is unlike what has been reported in the literature thus far.

## Case Reports

### Case 1

An 84-year-old Indian gentleman presented with a 1-month history of right eye pain, redness and photophobia. Four months earlier, he was diagnosed with stage 3 gastric adenocarcinoma (CPS <1; HER2 IHC 2+, FISH negative, pMMR). He had progressive gastric nodal spread despite having completed two cycles of Tegafur/Gimeracil/Oteracil (TS-1) adjuvant chemotherapy regimen 2 months prior. As he was not fit for surgery in view of his risks of receiving general anesthesia, a decision was made for him to receive treatment only with a palliative intent.

**Figure 1:**
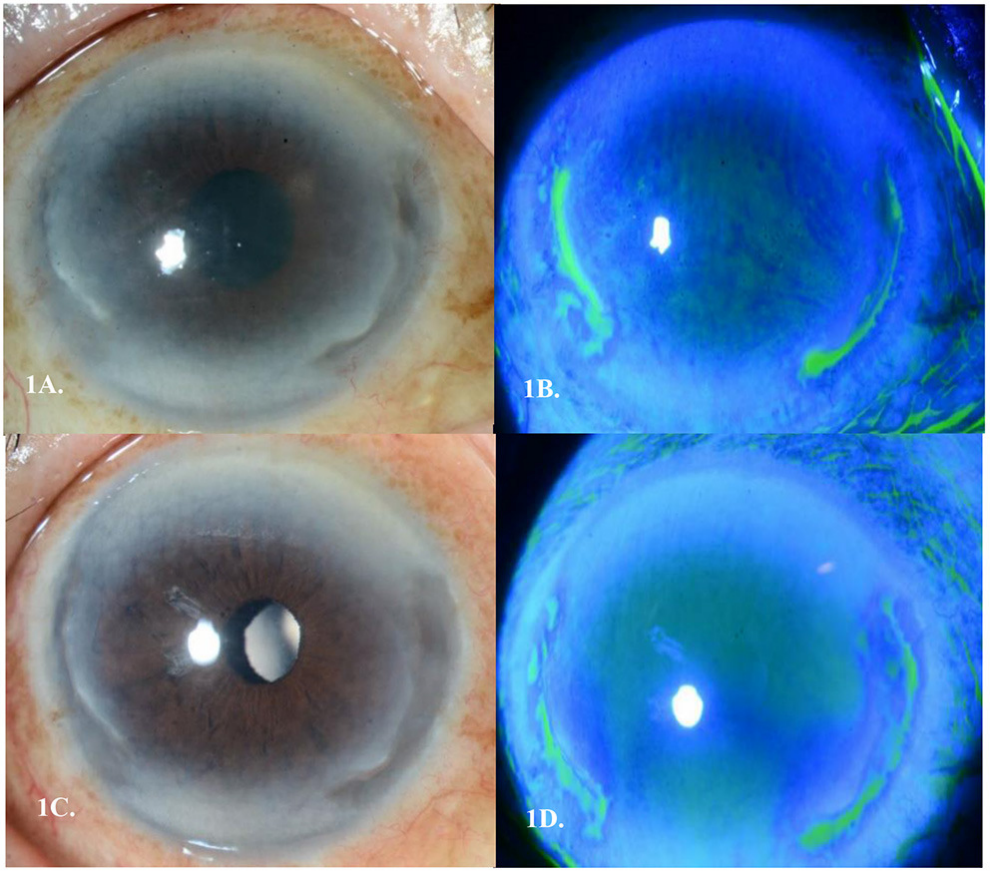
**(A)** Peripheral ulcerative keratitis (PUK) seen in Case 1's right eye with 2 discrete areas of severe peripheral corneal melt spanning 2 clock hours nasally and 2 clock hours temporally **(B)**. Corresponding staining of areas of cornea melt temporally and nasally **(C)**. Epithelization over the two areas of cornea melt by day 9 of treatment **(D)**. Corresponding area of pooling temporally and nasally showing epithelization of the stromal melt.

His symptoms of right eye redness and ocular discomfort started ~1 month after completing his chemotherapy. On presentation, his best corrected visual acuity (BCVA) was 6/18 in the right eye. Slit lamp examination revealed mild conjunctival injection with two discrete areas of severe peripheral corneal melt spanning two clock hours nasally and two clock hours temporally with 60% thinning. There was an associated overlying epithelial defect, with Descemet membrane folds, and the presence of anterior chamber cells (grade 2). There was no scleritis (Figures 1A,B). Left eye and posterior segmental examination of both eyes were unremarkable. Extensive autoimmune and infective work up, including a full blood count, tuberculosis and syphilis screen, and an autoimmune serum panel (antinuclear antibody, anti-double-stranded DNA antibody, antinuclear antibody profile, ANCA-EIA profile) all returned negative. Due to progressive severe corneal thinning, the patient was then started on IV Methylprednisolone 500 mg/OM for 3 consecutive days, as well as topical steroids of Prednisolone Acetate 1% (Pred Forte, Allergan, Irvine Ca) hourly, Levofloxacin 0.5% (Cravit^®^, Santen, Osaka, Japan) 3 hourly, oral Ciprofloxacin 500 mg twice daily, and oral Doxycycline 100 mg once daily. Over the course of 1 week, there was clinical improvement and thus his treatment was changed to oral Prednisolone 1 mg/kg that was tapered by 10 mg every 3 days. By day 9 of treatment, the area of corneal thinning had epithelized and there was no further stromal melting (Figures 1C,D).

### Case 2

**Figure 2:**
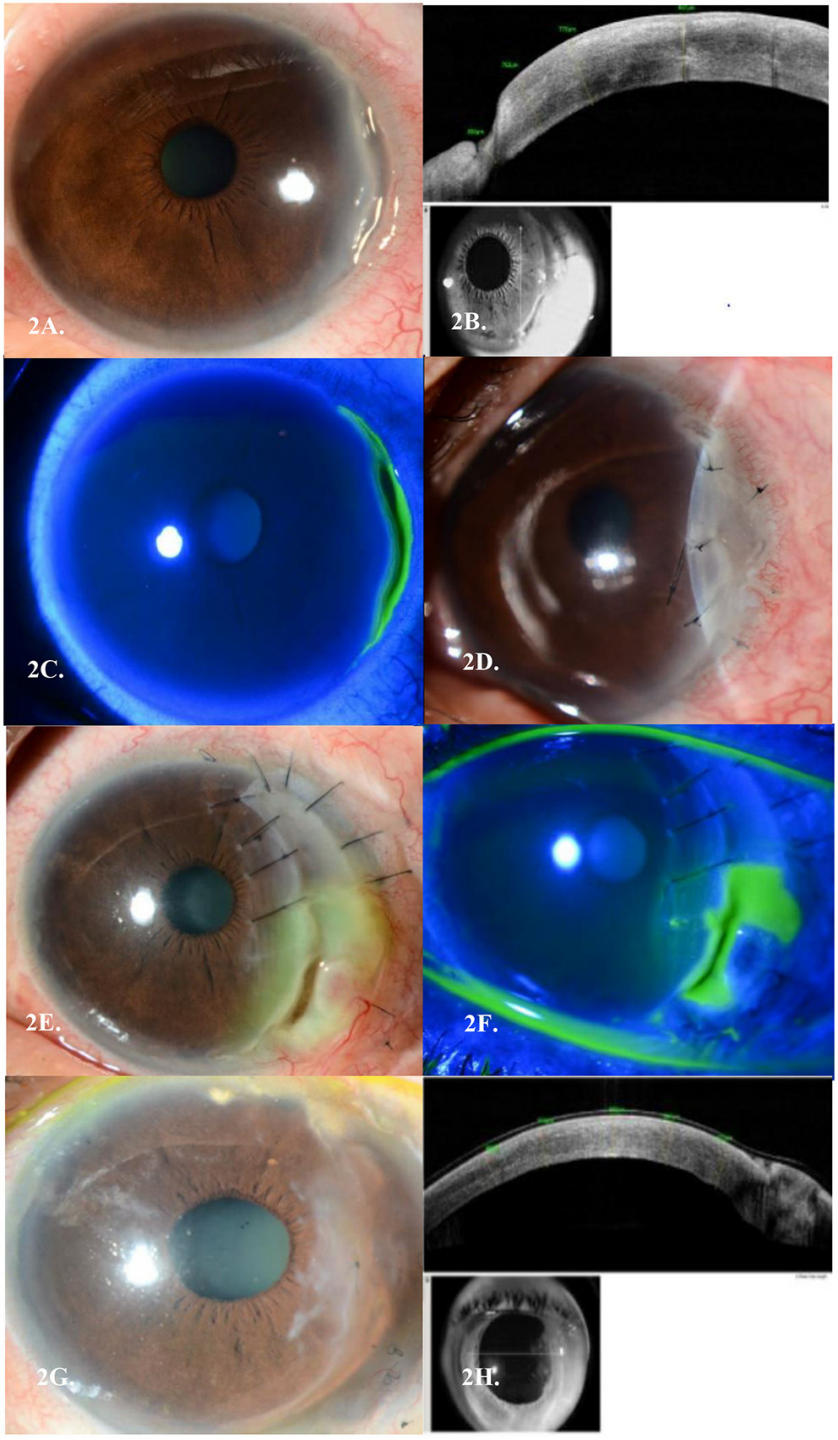
**(A)** Peripheral ulcerative keratitis (PUK) seen in Case 2's left eye temporally. **(B)** Corresponding AS-OCT cuts through the areas of thinning in each eye, respectively. **(C)** Corresponding staining of areas of cornea melt temporally. **(D)** Left Amniotic Membrane Transplant (AMT) in view of PUK progression. **(E)** Post repeat conjunctival recession, AMT and tectonic keratoplasty in view of PUK recurrences, with another new epithelial defect over the graft. **(F)** Corresponding staining of areas of cornea melt temporally over the tectonic graft. **(G)** Last AMT performed with no further recurrences of PUK over a 3-year follow-up period. **(H)** Corresponding AS-OCT cuts through the areas of thinning in each eye, respectively.

A 51-year-old Malay lady was referred for left eye redness and pain for 2 weeks. A previous diagnosis of right breast invasive ductal carcinoma (IDC) (ER/PR negative, but Herceptin 2 positive) was made more than 15 years ago and she had undergone wide excision and axillary clearance with adjuvant radiotherapy. Unfortunately, there was a recurrence of her right breast IDC 3 years prior, for which she then underwent a right complete mastectomy. Although she was offered adjuvant chemotherapy, she declined this treatment. On presentation, her BCVA was 6/6 in her left eye. Slit lamp examination of the left eye revealed an anterior stromal infiltrate with minimal thinning spanning 4 clock hours temporally and anterior chamber cells (grade 1). There was no scleritis (Figures 2A–C). Extensive autoimmune and infective work up was again performed which returned negative. She was started on topical Prednisolone Acetate 1% 4 times a day. A week later, the corneal thinning worsened to 50%, and she was started on oral Prednisolone 50 mg once a day. In view of the slow response to topical and oral steroids with progressive corneal thinning, she was given IV Methylprednisolone 500 mg once a day for 3 days. Despite this, her thinning progressed further to 80% and she underwent a left eye amniotic membrane transplant (AMT) (Figure 2D). She later needed further conjunctival recession, repeat amniotic membrane transplant and tectonic graft over the course of 2 years in view of repeated flares of her PUK with graft melting (Figures 2E,F). She was concurrently started on Mycophenolate Mofetil and Methotrexate which controlled her ocular inflammation. However, these immunosuppressants were stopped as she was not able to tolerate their systemic side effects. She was thus kept at a low dose of oral Prednisolone of 5 mg once daily. Her ocular condition remained quiescent with no further recurrences over a 3-year follow-up period (Figures 2G,H).

### Case 3

A 72-year-old Chinese lady presented with bilateral eye redness of an insidious onset. She had a history of multiple systemic malignancies known prior to her presentation. She was diagnosed with metastatic follicular thyroid carcinoma 13 years ago and had previous total thyroidectomy and radioactive iodine. This was complicated by lung and bony metastasis, which had been treated with radiotherapy 1 year prior to her ocular presentation. Furthermore, she had left breast IDC stage 1A (ER/PR positive CERB 2 negative) with previous mastectomy and axillary clearance a year prior. However, she declined chemotherapy and radiotherapy for this. More recently, she was diagnosed with a treatment-related myelodysplastic syndrome 3 months prior to her presentation to ophthalmic services and was started on subcutaneous recombinant erythropoietin (Erythropoietin beta, F. Hoffmann-La Roche, Basal, Switzerland). Her treatments for her malignancies were with a palliative intent. On presentation her BCVA was 6/18 in her right eye and 6/9 in her left eye. In her right eye, there was mild conjunctival injection with severe peripheral corneal melt of 60% thinning with an overlying epithelial defect spanning 2 clock hours nasally with minimal anterior chamber reaction, and in her left eye there was also peripheral 30% corneal thinning of 3 clock hours temporally and 1 clock hour inferiorly with the presence of anterior chamber cells (grade 2+). Posterior segment was unremarkable. Again, extensive autoimmune and infective work up, all returned negative. This patient was started on topical Prednisolone Acetate 1% hourly, Levofloxacin 0.5% 3 hourly, oral Ciprofloxacin 500 mg twice a day and Doxycycline 100 mg once a day. She subsequently developed active scleritis in both eyes. She was hence commenced on oral Prednisolone 50 mg once a day with a weekly taper of 10 mg. There was overall clinical improvement with epithelization over the area of thinning and cessation of further corneal melting upon commencement of her ocular therapies by day 10. Her corneal condition stabilized and scleritis resolved with the course of oral steroids. Unfortunately, the patient passed away 2 months later from metastatic complications.

## Discussion

In this paper, we report a case series of patients with PUK, as an ocular manifestation in the presence of a known underlying systemic solid organ malignancy. Our patients were all above 50 years old with underlying non-ocular malignancies. Two patients had metastatic diseases which failed chemotherapy or radiotherapy and were already on palliative care, while one patient had localized but recurrent disease that was in remission at the time of diagnosis.

**Figure 3:**
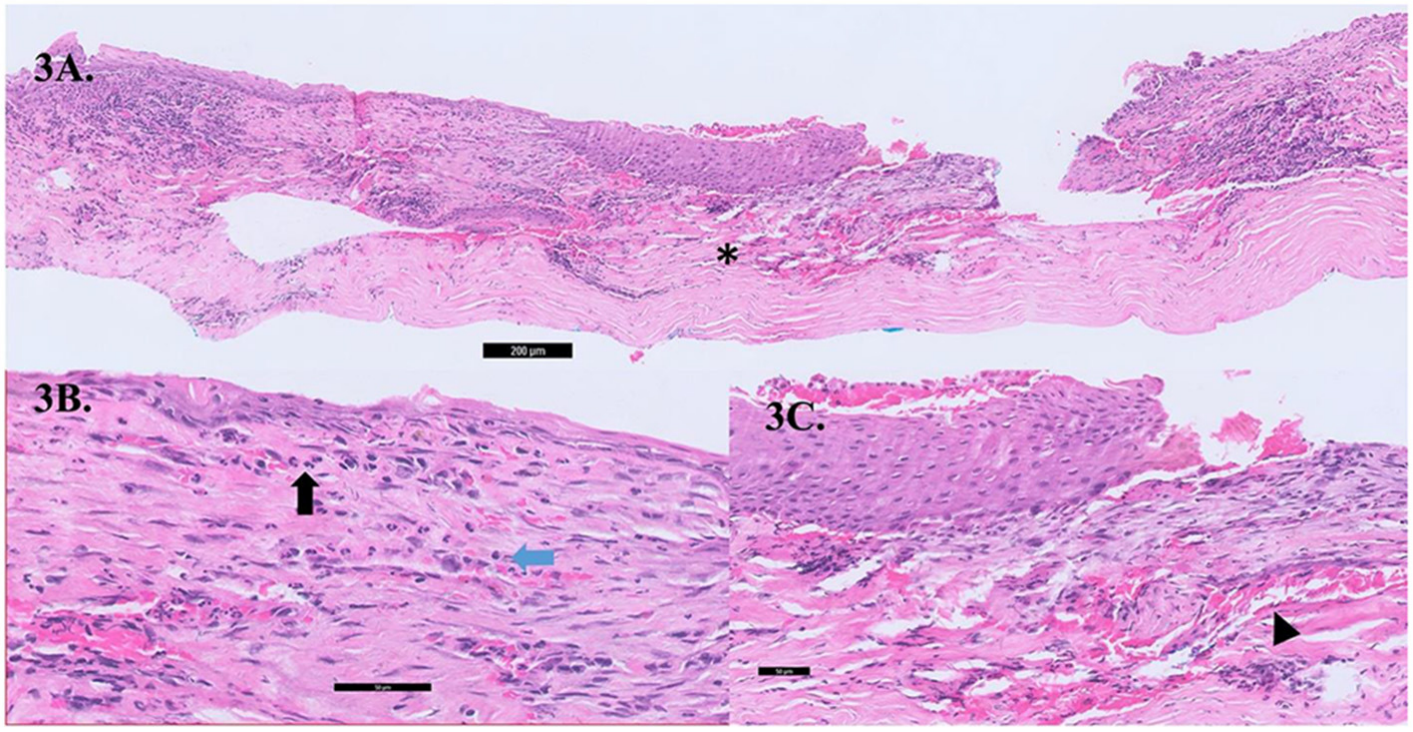
**(A)** Cross-section gross histology slide showing significant stromal thinning (asterix,*) with overlying epithelial overgrowth. **(B)** The conjunctival stroma showing acute on chronic inflammation comprising of lymphocytes (blue arrow) and neutrophils (black arrow) within the stroma with an absence of malignant cells. **(C)** Neovascularisation (black arrow head) seen within the corneal stroma.

We hypothesize that this could be related to a paraneoplastic process, where secreted autoantibodies from the distant tumor is channeled by blood vessels and lymphatics from adjacent conjunctiva, episclera, and sclera cross-reacts with corneal epithelial and stromal antigens and mediate destruction of the peripheral cornea (12). Similar inflammatory pathways are seen in PUK related to autoimmune diseases such as rheumatoid arthritis (13) or granulomatosis with polyangiitis (12, 14, 15). Histopathology evaluations of cornea biopsy specimens taken intraoperatively from Case 2 showed metaplastic epithelium with inflammatory infiltrates of lymphocytes and neutrophils within the stroma; no malignant cells were observed (Figures 3A–C). This implies that PUK in our patient is not likely due to direct malignant invasion. Earlier reports on PUK with associated malignancies were with newly diagnosed acute or chronic myeloid or lymphocytic leukemia (Table 1) (8–11). In contrast, in the acute exacerbation of CML or acute leukemia, leukemic blast cells are presumably deposited in the peripheral cornea by limbal conjunctival blood vessels that evoke inflammation and corneal thinning (8, 11). Various ocular anterior segment toxicities have been reported due to different systemic chemotherapy drugs; for instant, blepharitis, corneal thinning and melt associated with epidermal growth factor receptor inhibitors in the treatment of lung and colorectal cancer (16), and drug induced conjunctivitis due to BRAF inhbibitors such as pemetrexed and anthracyclines (17, 18). Only 1 of the 3 patients in our case series had been on a possible chemotherapy regimen (TS-1) that has been earlier reported to be associated with corneal epithelial disorders (19). Nonetheless, it is difficult to prove a casual relationship in this instance between his chemotherapy and his PUK, furthermore the patient had stopped the chemotherapy 2 months prior to his ocular presentation.

**Table 1:** Literature review on PUK associated with other malignancies.

**References**	**Age/gender**	**Underlying malignancy**	**Ocular outcome ocular medications**	**Systemic treatment**
Morjaria et al. (11)	74-year-old male	Newly diagnosed acute myeloid leukemia (AML)	Bilateral PUK – CF* vision PO prednisolone G. dexamethasone 6/9 (RE**) and 6/12 (LE^#^) after 2 weeks	Chemotherapy
Chawla et al. (10)	24-year-old female	Newly diagnosed acute lymphocytic leukemia (ALL)	Right eye PUK, scleritis and bilateral optic nerve infiltration – PL^∧^ vision G. Prednisolone acetate 1% G. Gatifloxacin 0.3%, G. Homatropine still PL (BE) after 4 weeks due to optic atrophy	Chemotherapy
Malecha et al. (8)	75-year-old male	“Blast crisis” of known chronic myelomonocytic leukemia (CMML) diagnosed 3 years prior	Right eye PUK and Left anterior uveitis – 6/30 (RE) and 6/9 (LE) G. Prednisolone acetate 1% G. HomatropinImproved over 3 weeks	Hydroxyurea and allopurinol passed away from CMML complications 5 months later
Sainz de la Maza et al. (9)	68-year-old male	Newly diagnosed chronic myelogenous leukemia	Bilateral PUK – 6/60 (BE) Had recurrence of PUK and needed conjunctival recession, keratectomy and cyanoacrylate application 6/9 (RE) and 6/21 (LE) at 9 months with no recurrences	Cyclophosphamide, switched to Hydrea (had RE PUK recurrence after), then switched back to cyclophosphamide
	67-year-old female	Contiguous sebaceous cell carcinoma of the upper eyelid and superior temporal and inferior orbit	Left eye PUK – HM vision Had conjunctival recession, keratectomy and cyanoacrylate application Underwent left globe exenteration, no metastatic spread	

*PUK, peripheral ulcerative keratitis; CF, counting fingers*.

^∧^Perception to light, *counting fingers, **right eye, #left eye.

From the earlier reports (8–11), most cases of newly diagnosed leukemia were detected promptly from the initial systemic work-up of PUK and had resolution with concurrent topical steroids and systemic chemotherapy for the underlying leukemia, except one case which had a delayed diagnosis of leukemia. That patient unfortunately required repeated conjunctival recessions, keratectomy and cyanoacrylate application in view a PUK recurrence (9). The cases of PUK in our series all required systemic steroids in addition to topical steroids, for which they had good responses, except Case 2 which required repeated AMTs, conjunctival recession surgeries, and second-line steroid-sparring immunosuppressive therapies. We postulate that the autoimmune response triggered by solid organ malignancies might require additional systemic immunosuppression compared to that of hematological malignancies that responded adequately to topical steroids, although this might be difficult to prove and requires further evaluations.

The two cases of treatment-responsive PUK (cases 1 and 3) had more advanced underlying metastatic disease that had failed chemotherapy treatment, with one of the cases having stopped TS-1 chemotherapy for his gastric adenocarcinoma 2 months prior to the diagnosis of his PUK. Case 2 had a more localized stage of her IDC that was amenable to surgical resection although she had refused adjuvant chemotherapy. Adjuvant chemotherapy is administered as standard of care due to the high rates of micro-metastases in breast carcinomas (20). This patient experienced recurrent PUK flares that required repeated surgical intervention over a course of 2 years, with no detectable recurrences of her PUK. These observations imply that paraneoplastic PUK could occur at any stage of the systemic malignancies and is not contingent with a progressive or more advanced stage of their underlying disease. It may well be that the serum autoantibodies persist for many years after the initial development of malignancy, causing an on-going immune reaction that leads to PUK recurrences despite a clearance of tumor burden. Nevertheless, serological testing of autoantibodies specific for paraneoplastic PUK has not been reported or investigated in the literature, unlike other posterior segment paraneoplastic disorders such as cancer-associated retinopathy, where the expression of Recoverin, a 23-kD protein, has been found to trigger an autoimmune attack on the retina (21). Further studies are still needed to understand the pathophysiology of the disease and optimization of their immunosuppressive regimen for this aggressive disease.

The treatment of paraneoplastic PUK in the setting of malignancy is very similar to that of PUK of any other etiologies. In all our cases, corneal tissue scrapings were performed initially to exclude any localized infection, and a infective and autoimmune work up was also carried out to exclude other inflammatory etiologies. Adequate lubrication, intensive topical steroids, ciclosporin, prophylactic antibiotics, and in severe cases systemic first and second-line immunosuppression were necessary to prevent worsening of the corneal melt. Surgical intervention in the form of conjunctival recession, AMT and tectonic keratoplasty was required, if there is a suboptimal response to immunosuppression and impending corneal perforation.

In conclusion, this case series highlights that, in addition to other infective or autoimmune causes, PUK may be the associated with life-threatening systemic solid tumor malignancies. We postulate that this may occur as a paraneoplastic phenomenon of the underlying malignancy. This case series highlights the necessity for clinicians to refer patients with known systemic malignancies who report ocular symptoms for an early ophthalmological review. This facilitates the detection of this sight-threatening disease, allowing for early therapeutic interventions and potentially better visual outcomes for these patients. Further research is needed to confirm the association of PUK as a paraneoplastic phenomenon in systemic malignancy, and to gain better insight into its pathophysiological mechanisms.

## Data Availability Statement

The raw data supporting the conclusions of this article will be made available by the authors, without undue reservation.

## Author Contributions

HO and VF were involved in the drafting and editing of the manuscript. JM and AC were involved in the editing of the manuscript. All authors contributed to the article and approved the submitted version.

## Conflict of Interest

The authors declare that the research was conducted in the absence of any commercial or financial relationships that could be construed as a potential conflict of interest. The handling editor Y-CL declared a shared affiliation with the author(s) at the time of review.

## Publisher's Note

All claims expressed in this article are solely those of the authors and do not necessarily represent those of their affiliated organizations, or those of the publisher, the editors and the reviewers. Any product that may be evaluated in this article, or claim that may be made by its manufacturer, is not guaranteed or endorsed by the publisher.
